# A specific neural substrate predicting current and future impulsivity in young adults

**DOI:** 10.1038/s41380-021-01017-0

**Published:** 2021-01-25

**Authors:** J. Scott Steele, Michele Bertocci, Kristen Eckstrand, Henry W. Chase, Richelle Stiffler, Haris Aslam, Jeanette Lockovich, Genna Bebko, Mary L. Phillips

**Affiliations:** 1 Department of Psychiatry, University of Pittsburgh, Pittsburgh, PA USA; 2 Brain Imaging Research Center, Department of Psychiatry, University of Arkansas for Medical Sciences, Little Rock, AR USA

**Keywords:** Neuroscience, Predictive markers

## Abstract

Impulsivity (rash action with deleterious outcomes) is common to many psychiatric disorders. While some studies indicate altered amygdala and prefrontal cortical (PFC) activity associated with impulsivity, it remains unclear whether these patterns of neural activity are specific to impulsivity or common to a range of affective and anxiety symptoms. To elucidate neural markers specific to impulsivity, we aimed to differentiate patterns of amygdala–PFC activity and functional connectivity associated with impulsivity from those associated with affective and anxiety symptoms, and identify measures of this circuitry predicting future worsening of impulsivity. Using a face emotion processing task that reliably activates amygdala–PFC circuitry, neural activity and connectivity were assessed in a transdiagnostically-recruited sample of young adults, including healthy (*N* = 47) and treatment-seeking individuals (*N* = 67). Relationships were examined between neural measures and impulsivity, anhedonia, and affective and anxiety symptoms at baseline (*N* = 114), and at 6 months post scan (*N* = 30). Impulsivity, particularly negative urgency and lack of perseverance, was related to greater amygdala activity (beta = 0.82, *p* = 0.003; beta = 0.68, *p* = 0.004; respectively) and lower amygdala–medial PFC functional connectivity (voxels = 60, *t*_peak_ = 4.45, *p*_FWE_ = 0.017; voxels = 335, *t*_peak_ = 5.26, *p*_FWE_ = 0.001; respectively) to facial fear. Left vlPFC, but not amygdala, activity to facial anger was inversely associated with mania/hypomania (beta = −2.08, *p* = 0.018). Impulsivity 6 months later was predicted by amygdala activity to facial sadness (beta = 0.50, *p* = 0.017). There were no other significant relationships between neural activity and 6-month anhedonia, affective, and anxiety symptoms. Our findings are the first to associate amygdala–PFC activity and functional connectivity with impulsivity in a large, transdiagnostic sample, providing neural targets for future interventions to reduce predisposition to impulsivity and related future mental health problems in young adults.

## Introduction

Young adulthood is a period of neural development and social transition, during which many mental health problems emerge [1], with nearly 18% of young adults seeking mental health services [2]. A behavioral problem common to this population is impulsivity, rash action especially in the context of positive or negative emotion-inducing contexts, which is implicated in numerous psychiatric disorders, particularly bipolar disorder, depressive disorders, ADHD, and borderline personality disorder [3–7]. By identifying objective neural markers of pathophysiologic processes underlying the development of impulsivity in young adulthood, neuroimaging studies can provide targets for new interventions to delay or even halt the neurodevelopmental processes that predispose to future psychiatric disorders in this critical developmental period.

During adolescence, subcortical emotion and reward circuity mature, with adolescents having heightened amygdala activity to emotional faces, but lower activity in emotional and behavioral regulatory regions in the ventral and dorsal prefrontal cortex (PFC) [8, 9]. Subsequently, during young adulthood, maturation of the PFC leads to improvements in emotional regulation and behavioral control [10, 11]. The medial PFC (mPFC), including ventromedial PFC (vmPFC), and rostral anterior cingulate cortex, regulates emotion-related activity in the amygdala [12], as does the ventrolateral PFC (vlPFC) [13], which is also involved in response inhibition [14, 15]. Yet, few neuroimaging studies focused on neural circuitry associated specifically with the development of impulsivity during this critical developmental period.

The UPPS-P Impulsive Behavior Scale is a comprehensive and well-validated measure of impulsivity [3, 4, 16], and includes five impulsivity subscales: negative and positive urgency—impulsive actions in the context of negative and positive emotion, which predispose to psychopathology [17], lack of premeditation, lack of perseverance, and sensation seeking. Neuroimaging studies of impulsivity predominantly employed the UPPS-P, and examined neural activity to emotional stimuli or during response inhibition. These studies reported a positive association between impulsivity and left amygdala and right vlPFC activity during passive viewing of negative emotional images [18]; greater dlPFC activity during response inhibition to negative emotional cues [19]; lower functional connectivity between the amygdala and vlPFC during emotional regulation [20]; and greater ventral striatal and left vlPFC activity during uncertain reward anticipation [21]. Additionally, right vlPFC activity during response inhibition was negatively associated with the UPPS-P negative urgency scale, but unrelated to the Barratt Impulsiveness Scale, another widely used impulsivity measure [22].

Together, these findings suggest a pattern of heightened subcortical, especially amygdala, activity to emotional stimuli, along with altered PFC activity and amygdala–PFC functional connectivity, as one potential mechanism underlying impulsivity. Sample sizes have been small, however, and few studies examined neural activity in relation to different impulsivity subscales, or adopted a transdiagnostic approach. Another limitation of the above studies is that other symptoms, such as anhedonia, and affective (depression, mania/hypomania) and anxiety symptoms also emerge in young adulthood [23], and are associated with altered amygdala and PFC activity [24–27]. For example, heightened amygdala activity, and aberrant PFC activity and amygdala–PFC connectivity, to emotional stimuli are common to mania/hypomania, depression, and anxiety, although these symptoms vary with regard to the specific PFC regions and emotional stimuli that evoke these responses (e.g., sadness in depression and threat in anxiety) [28–36]. By contrast, anhedonia is associated with lower amygdala and greater vmPFC activity to positive emotional stimuli and lower ventral striatal activity to positive emotional stimuli [26, 36, 37]. Thus, a large literature shows amygdala–PFC activity/connectivity alterations across impulsivity, anhedonia and affective and anxiety disorders. No studies to our knowledge, however, have examined the neural correlates specific to each of these behaviors and symptoms within a single design.

Recent neuroimaging studies identified neural marker predictors of future affective symptoms in adolescents [38, 39] and future changes in anhedonia in young adults [40]. Yet, to our knowledge, no studies attempted to identify neural marker predictors of future impulsivity. The overarching goal of the present study was thus to identify in young adults patterns of neural activity and functional connectivity associated specifically with impulsivity, and different impulsivity subscales, versus patterns of neural activity and functional connectivity that are common to impulsivity, anhedonia, affective and anxiety symptoms. We also wished to identify neural activity and functional connectivity predictors of increasing impulsivity severity, versus increasing anhedonia, affective and anxiety severity, over follow-up in young adults. For this purpose we examined a large sample of transdiagnostically-recruited young adults and examined activity of the neural regions implicated commonly across impulsivity, anhedonia, affective and anxiety symptoms: amygdala, vlPFC, and mPFC. We utilized a facial emotion processing task, known to activate emotion processing neural circuity across studies [41], and which has been found to specifically activate these structures in young adults [42].

We hypothesized that during an emotional face processing task:

1. Impulsivity would be related to greater amygdala and altered PFC activity, in particular vlPFC, to emotional stimuli, and lower functional connectivity between amygdala and PFC regions. While existing findings do not allow specific hypotheses regarding patterns of neural activity and connectivity that would be associated with different impulsivity subscales, prior studies suggest that relationships might be strongest for negative urgency.
2. The pattern of amygdala and PFC activity and functional connectivity associated with impulsivity, and specific impulsivity subscales in H1, would not be common to anhedonia, affective, or anxiety symptoms.
3. Patterns of amygdala and PFC activity and functional connectivity associated with baseline impulsivity would also predict future worsening of impulsivity 6 months later, and these findings would be specific to changes in impulsivity, rather than common to changes in anhedonia and affective and anxiety symptoms.

## Methods

### Participants and study design

One hundred and fourteen young adults ages 18–25 years were included in the final analyses (Table 1; Supplementary Information for power calculation). Participants were recruited via student counseling centers and advertisements in Pittsburgh as part of a larger study on the longitudinal course of affective and anxiety symptoms in young adults. One hundred and thirty-six participants had a neuroimaging assessment. Twenty-two participants (11 control, 11 distressed) were excluded due to excessive motion (>4 mm). Among the 114 participants in the final sample, 67 were seeking mental health treatment in the community for psychological distress and underwent assessment and scanning within 1 week of initial presentation. All participants were assessed for DSM-5 disorders via the Structured Clinical Interview for DSM-5 Disorders (SCID-5) [43]. No psychiatric diagnoses were exclusionary in this group, except active substance use disorder. At time of scan distressed participants could not be taking psychiatric medications, except those started within 2 weeks of scanning, although they could be started on any medication during the naturalistic follow-up period. Forty-seven healthy participants without current or past DSM-5 diagnosis or psychiatric medications were included to ensure that the entire sample represented the full range of symptoms and behaviors. Standard fMRI exclusionary criteria were applied (Supplementary Information). At baseline, all participants had a neuroimaging assessment and assessment by well-validated clinician-rated and self-report measures. Thirty participants within the distressed group were reassessed with repeated clinician-rated and self-report measures after a 6-month naturalistic follow-up period in which they could receive therapy and/or medication (Table 1).

**Table 1:** Demographic and clinical measures of the sample.

	Total	Healthy	Distressed baseline	Distressed baseline (subsample with 6-month follow-up)	Distressed 6-month follow-up	Distressed: 6-month vs. baseline significance testing
*N*	114	47	67	30	–	–
Age (y)	21.7 (2.1)	21.7 (2.1)	21.7 (2.1)	21.2 (1.73)	–	–
Gender (% female)	72%	72%	71%	83%	–	–
Education	5.4 (1.2)	5.6 (1.3)	5.4 (1.1)	5.2 (1.1)	–	–
UPPS-P total	2.1 (0.38)	1 (0.33)	2.2 (0.38)	2.1 (0.32)	2.1 (0.38)	−0.7322 (*p* = 0.47)
Negative urgency	2.2 (0.62)	1.8 (0.48)	2.5 (0.51)	2.5 (0.57)	2.3 (0.56)	−1.38 (*p* = 0.17)
Lack of premeditation	1.8 (0.45)	1.7 (0.35)	1.8 (0.52)	1.7 (0.47)	1.7 (0.45)	−0.03 (*p* = 0.98)
Lack of perseverance	2.0 (0.54)	1.7 (0.43)	2.2 (0.51)	2.2 (0.56)	2.2 (0.60)	−0.25 (*p* = 0.81)
Sensation seeking	2.8 (0.58)	3.0 (0.53)	2.7 (0.55)	2.5 (0.58)	2.6 (0.70)	0.48 (*p* = 0.63)
Positive urgency	1.7 (0.61)	1.6 (0.45)	1.9 (0.65)	1.8 (0.53)	1.6 (0.61)	−1.0 (*p* = 0.32)
MASQ-AD	2.9 (0.71)	2.4 (0.58)	3.3 (0.55)	3.3 (0.60)	2.9 (0.66)	**−2.38 (*p*** = **0.02)**
YMRS	1.5 (1.9)	0.3 (1.4)	2.4 (1.7)	2.4 (1.57)	2.6 (2.25)	0.27 (*p* = 0.79)
HAM-D	8.9 (7.7)	1.4 (2.3)	14.2 (5.4)	13.8 (5.50)	11.4 (5.3)	−1.72 (*p* = 0.09)
HAM-A	7.3 (6.7)	1.0 (1.2)	11.8 (5.2)	11.0 (5.15)	9.0 (5.6)	−1.41 (*p* = 0.16)
MDD	45%	0%	76%	77%	–	–
GAD	46%	0%	79%	83%	–	–
Bipolar disorder	2.6%	0%	4.5%	3.3%	–	–
ADHD	3.5%	0%	6.0%	6.7%	–	–
Taking medication	2.6%	0%	4.5%	0%	20%	**6.67 (*p*** = **0.01)**

Mean and SD reported for clinical measures. Age, gender, education, and diagnoses were assessed at time of fMRI scan and not repeated at 6 months. Education level according to University of Pittsburgh Demographic form and definitions of levels are provided in Supplementary Information. Participants in distressed group may be diagnosed with more than one disorder. See Supplementary Information for specific medication regimens of participants. Significance testing between baseline and 6-month follow-up for the distressed sample with 6-month follow-up data. T statistic and *p* value reported for testing of continuous variables; chi-squared statistic and *p* value reported for testing of proportion on medication.

The University of Pittsburgh Institutional Review Board approved the study and participants provided written informed consent.

### Impulsivity, anhedonia and affective and anxiety measures

Impulsivity severity was assessed using the UPPS-P Impulsive Behavior Scale [3, 4]. This scale consists of 59 questions using a Likert scale to assess 5 impulsivity subscales, which are summed to provide total score. Anhedonia was assessed using the Anhedonic Depression subscale of Mood and Anxiety Symptom Questionnaire (MASQ-AD), which discriminates anhedonic depression from general distress [44]. To test for relationships with depression, anxiety, and mania/hypomania participants were administered the Hamilton Depression Scale (HAM-D) [45], Hamilton Anxiety Scale (HAM-A) [46], and Young Mania Rating Scale (YMRS) [47]. The assessments of impulsivity and anhedonia were self-report measures and scales of depression, anxiety, and mania/hypomania were clinician rated. Although we did not selectively recruit patients with bipolar disorder, subthreshold hypomania symptoms are important to examine as they are predictive of future bipolar disorder [48]. Among the 30 distressed participants with 6-month follow-up data, statistical significance of differences between baseline and 6 months was tested using a two sample *t* test; chi-squared test was used for proportion on medication.

### fMRI region of interest activity and functional connectivity

The dynamic faces task [49] was used to measure emotion-related activity in a mask of emotion processing/regulation neural circuity. Details regarding fMRI task, data acquisition, preprocessing, and first level analyses are available in Supplementary Information.

### H1 and H2. Neural activity associated with baseline symptom and behavior measures

We used elastic net penalized least squares regression for variable selection (implemented via R GLMNET). Elastic net can be used with correlated predictors and is robust to GLM assumption violations [50]. Parameter estimates from neural regions identified in first level analysis and demographic variables (age/gender/education level) were included as independent variables; the dependent variable was one of the five symptoms (UPPS-P/MASQ-AD/HAM-D/YMRS/HAM-A) in five parallel elastic net models, thus determining nonzero coefficients associating independent variables (neural activity) with dependent variable (symptom severity).

Identified nonzero coefficients from the elastic net models were then tested for statistical significance using linear robust regression, an iteratively reweighted least squares regression that protects against the influence of outliers and yields better detection with lower false positives for group level fMRI analyses [51, 52]. In each of the five parallel models, regression was performed while controlling for demographic variables that also survived variable selection for that model. A significant relationship with UPPS-P total prompted examination of relationship with each UPPS-P subscale.

The False Discovery Rate (FDR) controlled for multiple parallel tests, using the Benjamini–Yekutiele FDR method [53]. There is overlap between information captured on self-report measures of impulsivity and anhedonia and clinician-rated measures of depression, mania, and anxiety. However, self-report measures capture additional information about symptoms that is not measured by clinician-rated measures [54]. Therefore, to allow family-wise assessment of like assessment methods, we grouped these two sets of measures separately. We performed one FDR correction for all nonzero coefficients for the self-report measures (UPPS-P/MASQ-AD) and a second FDR correction for nonzero coefficients for clinician-rated scales (YMRS/HAM-D/HAM-A). When examining the 5 UPPS-P subscales, we adjusted the statistical threshold for five parallel tests.

In secondary analyses, given a priori expectations of alterations in amygdala–PFC connectivity, we examined relationships among functional connectivity between the amygdala and the rest of the mask for those task conditions in which amygdala activity was associated with symptoms. Functional connectivity was calculated using Generalized Psychophysiological Interaction (gPPI) [55, 56]. Regression of functional connectivity with impulsivity was performed in SPM12, controlling for demographic variables (age/gender/education). We used anatomically-defined amygdala seeds defined by the WFU Pickatlas, and a one sample *t* test. A significance threshold of *p* < 0.001 uncorrected with minimum cluster size 30 voxels was used to threshold results and only clusters with whole mask FWE-adjusted *p* values of < 0.05 are reported [57, 58].

### H3. Neural activity predicting behavioral measures 6 months later

In the clinical subpopulation with 6-month follow-up data, we used an analogous approach to H1 and H2 analyses, using elastic net followed by linear robust regression. In these models, medication load change (6-month minus baseline) was included as a covariate along with demographic variables. Psychotropic medication load is a standardized measure across medication types and has been previously used in neuroimaging studies to capture information about medication status when lacking adequate power to examine specific medication effects [59, 60] (Supplementary Information for calculation and medication regimens). We predicted 6-month severity while including baseline severity as a covariate [61]. Analyses were grouped for FDR correction as in baseline analyses.

In secondary analyses, analogous to H1 and H2, we used gPPI to examine relationships among 6-month symptoms and functional connectivity between the amygdala and the rest of the mask, covarying for baseline symptoms, and controlling for demographic variables.

### Sensitivity analyses

Given high levels of impulsivity in individuals with bipolar disorder [62, 63], this subgroup might disproportionately drive our main findings. Thus, in post hoc sensitivity analyses we excluded this subgroup and also controlled for MDD, GAD, and ADHD diagnoses (yes/no) to test for impact of diagnosis on findings. To test for impact of medication status at baseline we removed participants taking medication.

### Specificity of findings

Post hoc, to assess the specificity of significant associations of neural measures with symptoms in the main analysis, we repeated the significant analyses substituting the remaining symptoms as the dependent variable in parallel analyses. This was done using linear regression predicting *z*-scored symptom measures, allowing direct comparison of the 95% confidence intervals of the beta values for each symptom.

## Results

See Table 1 for clinical characteristics of participants.

### Task-related activity

Left and right amygdala showed significant activity to each facial emotion; the left vlPFC and left vmPFC showed significant activity to facial anger and fear; the left vmPFC showed significant activity to facial sadness (Supplementary Table 1). None of the regions showed significant activity to shape > emotion.

### H1 and H2

#### Impulsivity and anhedonia

**Fig. 1:**
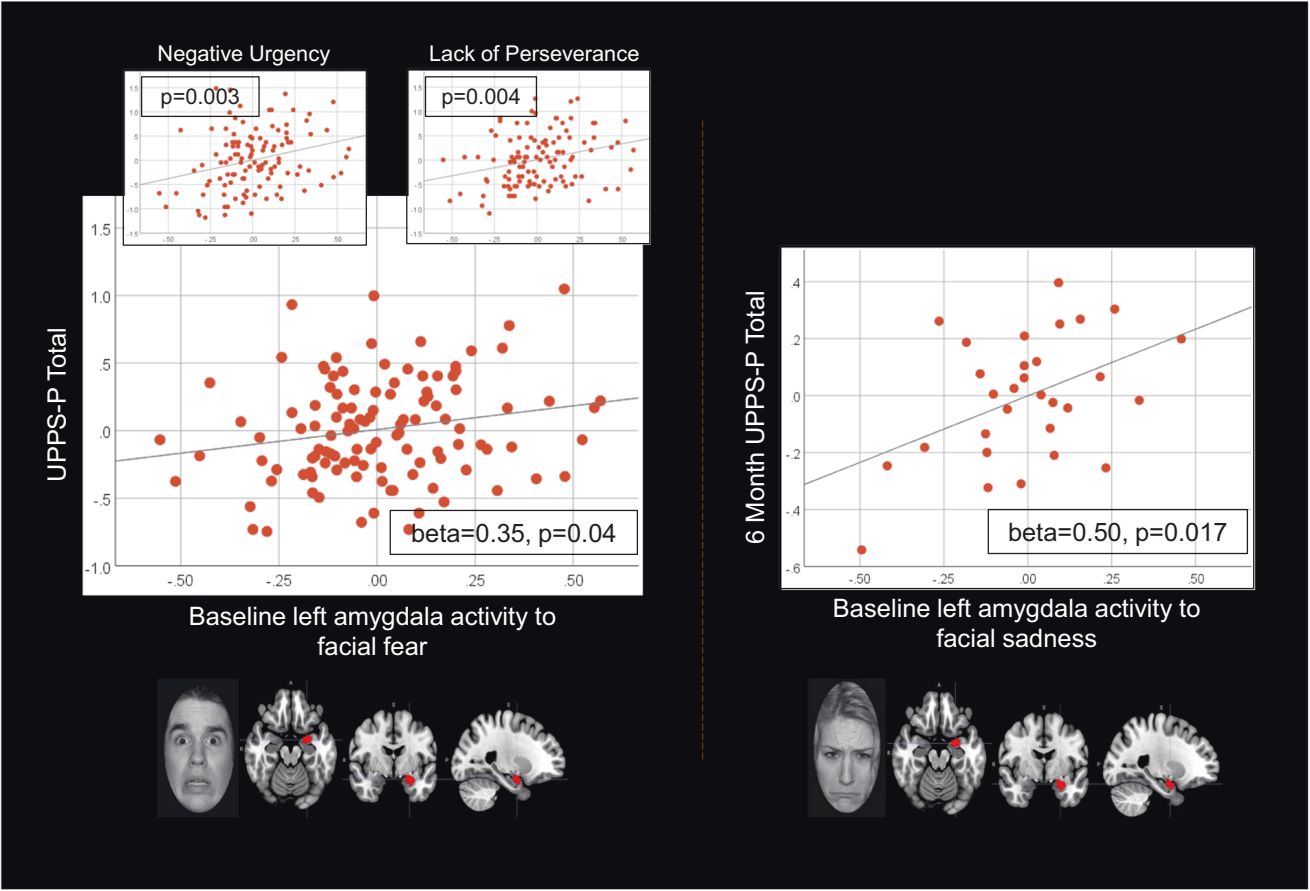
Left amygdala activity predicted present and future impulsivity. The left vertical panel depicts relationships between left amygdala activity to facial fear and UPPS-P total (beta = 0.35, *p* = 0.04), and between left amygdala activity to facial fear and negative urgency (beta = 0.82, *p* = 0.003) and lack of perseverance (beta = 0.68, *p* = 0.004). The right panel depicts impulsivity at 6 months predicted by left amygdala activity to facial sadness at baseline (beta = 0.50, *p* = 0.017). Partial regression plots of residuals are used to depict relationships from multiple regession models (covarying for gender in the left panel and baseline impulsivity in the right panel).

Elastic net analysis revealed one nonzero coefficient between UPPS-P total and neural activity: left amygdala activity to facial fear, and gender (Table 2). There were no nonzero coefficients between neural activity measures and MASQ-AD. Subsequent regression (using a 1 test FDR threshold: *p* = 0.05), and controlling for gender, revealed left amygdala activity to facial fear was significantly associated with UPPS-P total score (beta = 0.3458, *p* = 0.0406; Table 2; Fig. 1). Examination of UPPS-P subscales, controlling for gender (five test FDR threshold: *p* = 0.0219), revealed left amygdala activity to facial fear was related to negative urgency (beta = 0.8237, *p* = 0.0028) and lack of perseverance (beta = 0.6831, *p* = 0.004; Table 2; Fig. 1).

In sensitivity analyses, excluding bipolar disorder and controlling for diagnoses (MDD/GAD/ADHD) the relationship between amygdala activity to facial fear and UPPS-P total score was no longer significant (*p* = 0.15) (Supplementary Table 2). Excluding participants taking medication, reduced the finding to *p* = 0.0581 (Supplementary Table 3). However, relationships among left amygdala activity to facial fear and negative urgency and lack of perseverance remained significant when controlling for diagnosis and removing participants with bipolar disorder (five test FDR threshold: *p* = 0.0219): beta = 0.6069, *p* = 0.010; beta = 0.6700, *p* = 0.003, respectively, and removing participants taking medication (five test FDR threshold: *p* = 0.0219): beta = 0.8374, *p* = 0.003; beta = 0.6528, *p* = 0.007, respectively (Supplementary Tables 4, 5).

#### Clinician-rated symptoms

No nonzero coefficients were observed for relationships between neural activity measures and HAM-D or HAM-A. Nonzero relationships were observed between YMRS and four neural activity measures and education (Table 2). Controlling for education, one neural measure, left vlPFC activity to facial anger, reached statistical significance in regression analyses (four test FDR threshold: *p* = 0.024): beta = −2.08, *p* = 0.018. When controlling for diagnoses, this reduced to trend-level significance (beta = −1.13, *p* = 0.052), but remained significant when removing participants taking medication (beta = −2.1215, *p* = 0.016; Supplementary Tables 6, 7).

**Table 2:** Predicting baseline symptoms/behavior from neural measures.

	UPPS-P total	MASQ-AD	YMRS	HAM-D	HAM-A	Negative urgency	Lack of premeditation	Lack of perseverance	Sensation seeking	Positive urgency
Gender	0.17 (0.043)	*	*	*	*	−0.13 (0.33)	−0.02 (0.86)	0.18 (0.11)	0.30 (0.02)	0.22 (0.084)
Age	*	*	*	*	*					
Education	*	*	−0.30 (0.072)	*	*					
R Amygdala anger	*	*	*	*	*					
L Amygdala anger	*	*	*	*	*					
L vlPFC anger	*	*	***−2.08 (0.018)***	*	*					
L vmPFC anger	*	*	*	*	*					
R Amygdala fear	*	*	*1.13 (0.16)*	*	*					
L Amygdala fear	***0.35 (0.041)***	*	*	*	*	***0.82 (0.003)***	*0.27 (0.17)*	***0.68 (0.004)***	*−0.14 (0.59)*	*0.25 (0.33)*
L vlPFC fear	*	*	*	*	*					
L vmPFC fear	*	*	*0.38 (0.46)*	*	*					
R Amygdala sad	*	*	*	*	*					
L Amygdala sad	*	*	*−1.47 (0.057)*	*	*					
L vmPFC sad	*	*	*	*	*					
R Amygdala happy	*	*	*	*	*					
L Amygdala happy	*	*	*	*	*					

Beta and FDR adjusted *p* values in linear regression models are reported for nonzero coefficients from the elastic net regression models. Asterisks indicate zero coefficients from the elastic net regression models. Each of the five impulsivity/other symptom linear regression models was run separately. FDR correction for multiple tests was performed across self-report (impulsivity and anhedonia) and clinician-rated symptoms (mania/hypomania, depression, and anxiety) (indicated by asterisks and values given in italics). Each neural measure was tested separately, controlling for demographic variables. For significant relationships between neural activity and UPPS-P total score, relationships with UPPS-P subscales were examined, controlling for those demographic variables that were related to UPPS-P total score.

#### Secondary functional connectivity analyses

**Fig. 2:**
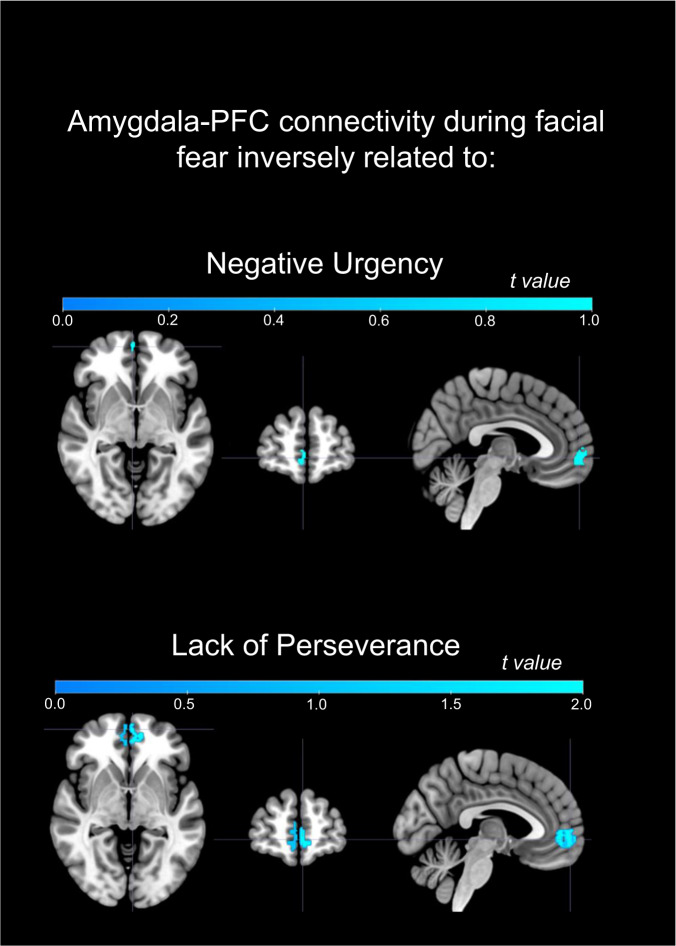
Inverse relationship between amygdala–mPFC functional connectivity to facial fear > shape and impulsivity measures. Only significant clusters are shown meeting threshold *p*_uncorrected_ < 0.001, *k* ≥ 30.

Functional connectivity between the amygdala–mPFC (Brodmann Area 10) to facial fear was significantly inversely related to negative urgency (voxels = 60, *t*_peak_ = 4.45, *p*_FWE_ = 0.017) and lack of perseverance (voxels = 335, *t*_peak_ = 5.26, *p*_FWE_ = 0.001; Fig. 2). Removing participants taking medication and controlling for mood/anxiety/ADHD diagnoses, lack of perseverance remained inversely related to amygdala–mPFC connectivity (voxels = 67, *t*_peak_ = 4.19, *p*_FWE_ = 0.043), although negative urgency did not.

### H3

#### Impulsivity and anhedonia

Nonzero coefficients were observed for relationships between UPPS-P total score at 6 months and left amygdala activity to facial sadness and happiness, and left vlPFC activity to facial anger; nonzero coefficients were also observed between MASQ-AD at 6 months and left amygdala activity to facial sadness and anger, left vlPFC activity to facial anger, and left vmPFC activity to facial anger and sadness (Table 3). No demographic variables or medication load had a nonzero relationship with 6-month impulsivity or MASQ-AD. In regression analyses (baseline UPPS-P total score and anhedonia score as covariates, above eight neural measure independent variables: ten test FDR threshold: *p* = 0.017), there was a significant relationship between left amygdala activity to facial sadness and 6-month UPPS-P total score (beta = 0.50, *p* = 0.017; Table 3). This reduced to trend level when controlling for diagnoses (ten test FDR threshold: *p* = 0.017): beta = 0.45, *p* = 0.037 (Supplementary Table 8), and when controlling for medication load change (ten test FDR threshold: *p* = 0.017): beta = 0.51, *p* = 0.020 (Supplementary Table 9). Regression of relationships between amygdala activity to facial sadness and 6-month UPPS-P subscales revealed no significant relationships. Regression predicting 6-month MASQ-AD found no significant relationships (Table 3).

**Table 3:** Predicting 6-month symptoms/behavior controlling for baseline scores.

	UPPS-P total	MASQ-AD	YMRS	HAM-D	HAM-A	Negative urgency	Lack of premeditation	Lack of perseverance	Sensation seeking	Positive urgency
Gender	*	*	*	*	*					
Age	*	*	−0.62 (0.25)	*	*					
Education	*	*	*	*	*					
Medication load change	*	*	*	*	*					
Baseline symptoms/behaviors	*0.98 (0.0000)*	*0.73 (0.0002)*	*0.37 (0.18)*	*0.59 (0.001)*	*0.53 (0.002)*	*0.83 (0.0000)*	*0.70 (0.0000)*	*0.98 (0.0000)*	*1.11 (0.0000)*	*0.91 (0.0000)*
R Amygdala anger	*	*	*	*	*−9.7 (0.057)*					
L Amygdala anger	*	*0.75 (0.18)*	*	*	*					
L vlPFC anger	*	*0.80 (0.16)*	*	*	*					
L vmPFC anger	*	*0.47 (0.12)*	*	*	*					
R Amygdala fear	*	*	*−4.7 (0.055)*	*	*−9.5 (0.081)*					
L Amygdala fear	*	*	*	*	*					
L vlPFC fear	*−0.34 (0.27)*	*	*	*	*					
L vmPFC fear	*	*	*	*	*					
R Amygdala sad	*	*	*	*	*					
L Amygdala sad	***0.50 (0.017)***	*0.55 (0.23)*	*	*	*	*0.41 (0.17)*	*0.049 (0.86)*	*0.51 (0.098)*	*0.34 (0.27)*	*0.38 (0.29)*
L vmPFC sad	*	*0.50 (0.078)*	*	*	*					
R Amygdala happy	*	*	*	*	*					
L Amygdala happy	*0.30 (0.025)*	*	*	*	*					

Beta and FDR adjusted *p* values in linear regression models are reported for nonzero coefficients from the elastic net regression models. Asterisks indicate zero coefficients from the elastic net regression models. Each of the five impulsivity/other symptom models was run separately. FDR correction for multiple tests was performed across self-report (impulsivity and anhedonia) and clinician-rated symptoms (mania/hypomania, depression, and anxiety) (indicated by asterisks and values given in italics). Each neural measure was tested separately controlling for demographic variables. For significant relationships between neural activity and UPPS-P total score, relationships with UPPS-P subscales were examined, controlling for those demographic variables that were related to UPPS-P total score.

#### Clinician-rated symptoms

Although the elastic net models identified nonzero relationships between three neural measures and clinician-rated symptoms at 6 months, none of these reached statistical significance in linear robust regression analyses (FDR corrected significance threshold for three neural measures + three baseline symptom measures: six tests = *p* = 0.0204; Table 3).

#### Secondary functional connectivity analyses

Predicting 6-month impulsivity with functional connectivity of the amygdala related to facial sadness found no clusters in the mask meeting significance threshold.

#### Specificity of findings

See Supplementary Information section specificity of findings Results and Supplementary Tables 10–13.

## Discussion

The goal of the present study was to increase understanding of the neural mechanisms associated with present and future impulsivity, and the extent to which these processes are specific to impulsivity rather than common to anhedonia and affective and anxiety symptoms. Among a large and heterogeneous sample of young adults, baseline greater impulsivity was associated with greater amygdala activity to facial fear. This pattern of amygdala activity was specifically related to negative urgency and lack of perseverance, and these impulsivity subscales were associated with lower functional connectivity between the amygdala and the anterior mPFC. Additionally, amygdala activity to facial sadness positively predicted greater impulsivity 6 months later. Baseline and future anhedonia and clinician-rated measures of affective and anxiety symptoms were not significantly associated with neural activity.

Our findings support those of prior smaller studies of the neural correlates of impulsivity, which generally reported greater amygdala activity to threat-related stimuli, and lower connectivity between the amygdala and prefrontal cortices [18, 19]. We show that these finding generalize across a large and heterogeneous sample, including young adults seeking treatment for psychological distress and control participants, and are linked most strongly with negative urgency and lack of perseverance. Greater amygdala activity to facial fear likely reflects greater attention to threat, and lower functional connectivity between the amygdala and the anterior mPFC, specifically BA10, an inability to plan future actions in negative emotional contexts, given the role of BA10 in action planning [64, 65]. Thus, the combination of these patterns of activity and functional connectivity is a potential neural mechanism predisposing to impulsive behaviors triggered in negative emotional contexts, i.e., negative urgency, and lack of an ability to plan, i.e., lack of perseverance.

At 6 months only impulsivity was predicted by neural activity: left amygdala activity to facial sadness, an important social facial emotion, and not by facial fear. While the role of the amygdala in the response to environmental threat is well-established [66, 67], the amygdala is also a critical component of neural circuits important for avoidance of distress-inducing negative emotional contexts [68, 69]. Thus, in contrast to higher levels of amygdala activity to environmental threat indicated by facial fear, which might predispose to acute threat-related impulsive behavior, reflected by higher levels of negative urgency and lack of perseverance, greater amygdala activity to facial sadness might predispose to increasing levels of social distress-related impulsive avoidance behaviors, reflected by future worsening of general impulsivity.

The absence of significant relationships between anhedonia and neural activity was unexpected. Although affective images have been used effectively to study anhedonia in specific populations [26, 36, 37], one consideration is whether these results generalize to a transdiagnostic sample as facial expressions are likely less intrinsically rewarding or pleasurable than actual rewards. Thus, reward processing paradigms might be more relevant to study neural mechanisms of anhedonia, and have been used effectively in transdiagnostic samples [40]. One neural activity measure, left vlPFC activity facial anger, was inversely related to baseline mania/hypomania. This relationship might reflect a lack of regulation of the amygdala by the vlPFC, paralleling findings from previous studies of bipolar disorder [70–72].

The post hoc tests examining the specificity of our main findings to impulsivity and mania/hypomania revealed that while these results were strongest for impulsivity and mania/hypomania, relationships were also present between neural activity and other symptom measures, although at nonsignificant levels with overlapping 95% CI. These additional analyses did reveal, however, that left amygdala–mPFC FC to facial fear at baseline was associated with self-reported anhedonia and clinician-rated depression, anxiety, and mania/hypomania. Thus, while activity findings were largely specific to impulsivity measures and FC changes appear to be less specific, future studies are needed to clarify the extent to which these neural measures are associated with other symptom measures. While impulsivity is a component of several of these symptoms, especially mania/hypomania, the latter comprise a much broader range of behaviors. Mania/hypomania, for example, includes changes in sleep and energy levels, and disorganization/dysregulated behaviors that might be more closely linked to activity in other neural circuitries [73], including a general lack of emotional regulation, as indicated by the lower vlPFC activity finding above. Given that impulsivity is a key component of mania/hypomania, however, identifying specific neural markers of impulsivity is an important step toward elucidating key pathophysiologic processes underlying vulnerability to mania/hypomania and complex psychiatric symptoms and behaviors.

Limitations of the present study are that although this is one of the largest neuroimaging studies of impulsivity, replication in independent samples is required, especially for longitudinal, prospective analyses. Additionally, as a naturalistic follow-up, participants were taking medication, but only 3 out of 114 were taking medication at baseline, and fewer than a quarter of participants started or changed medication during follow-up. Future studies can examine effect of specific treatments on impulsivity-related neural circuitry. While we observed few relationships with gender, future studies can focus more specifically on gender effects.

Our present findings are the first to associate amygdala–prefrontal cortical activity and functional connectivity with specific impulsivity subscales in a large, transdiagnostic sample, and that amygdala activity to facial emotion predicts future worsening impulsivity. Our findings identify a potential neural mechanism for predisposition to impulsivity in young adults, which can provide neural measures to act as targets for, and to monitor the effectiveness of, future interventions aiming to reduce predisposition to impulsive behaviors and future mental health problems in young adults.

## Supplementary information

Supplemental Information
