# Supplementary material for: A specific neural substrate predicting current and future impulsivity in young adults
Source: Mol Psychiatry. 2021 Jan 25;26(9):4919–30. doi: 10.1038/s41380-021-01017-0 (PMC8589683; doi:10.1038/s41380-021-01017-0)
Supplement: Supplementary file 1 — Supplemental Information [file 41380_2021_1017_MOESM1_ESM.doc]

Supplemental Information

Power Analysis

Expected medium effect size f^2^=0.15 for brain-behavior relationships, and p=0.001, power = 0.83, N=114.

Participant Exclusion Criteria

Exclusion criteria included: a visual disturbance (<20/40 Snellen visual acuity) when corrected by glasses; presence of metallic foreign objects in body, such as aneurysm clips or pacemakers, or a questionable history of metallic fragments; positive pregnancy test for female individuals, or self-reporting of pregnancy; claustrophobia; a Mini-Mental State Examination score <24; a premorbid IQ estimate <85 (as determined by the National Adult Reading Test); presence of an alcohol, tobacco, or substance use disorder in the prior 3 months; current treatment with psychotropic medication for >2 weeks; previous psychotropic medication treatment in the past 6 months; any history of serious medical/physical conditions: neurological disorder, history of brain tumor/brain surgery, progressive endocrine disorder, heart disorder, or other major systemic medical conditions (kidney disease, multiple sclerosis, cerebral palsy, blindness, serious physical disability) or chronic/acute condition including any managed by medication (chronic back problem, recent surgery); taking medication for an excluded medical condition. For healthy participants, personal history of any psychiatric disorder or psychotropic medication use were also exclusion criteria.

Psychotropic Medication Load

Psychotropic medication load is a measure of the number and dosages of medications for each participant^1-2^. Mood stabilizers and antidepressants were converted to low- and high-dose groups, with low-dose coded as levels 1 and 2 and high-dose as 3 and 4, based on previously used criteria. Participants taking no medication were coded as 0. Antipsychotics were converted to chlorpromazine dose equivalents with low- and high-dose, 1 and 2 respectively, representing chlorpromazine equivalents dose equal or below, or above, mean effective daily dose of chlorpromazine. Benzodiazepine dose was coded as 0, 1 or 2, with reference to the midpoint of the Physician’s Desk Reference-recommended daily dose range for each medication. A composite measure of psychotropic medication load was calculated by summing the individual medication codes for each medication category for each individual participant. The change in psychotropic load between baseline and 6-month follow-up was calculated as the mean difference in psychotropic medication load between timepoints.

Psychiatric medication regimens and equivalent med load for those participants taking medication (N/A indicates data not available due to lack of follow up):

| Participant | Med load | Baseline | 6 months |
| --- | --- | --- | --- |
| A | 2 | Lithium 1200mg daily | N/A |
| B | 1 | Bupropion 150mg daily | N/A |
| C | 1 | Mirtazapine 10mg | N/A |
| D | 2 | None | Paroxetine 30mg |
| E | 1 | None | Bupropion 300mg |
| F | 1 | None | Bupropion 150mg |
| G | 1 | None | Trazodone 50mg |
| H | 2 | None | Sertraline 150mg |
| I | 1 | None | Sertraline 125mg |

Emotional Faces Task Design

The emotional faces task was administered during fMRI scanning and lasted 12.5 minutes. Stimuli were faces from the NimStim dataset and morphed in 5% increments from neutral (0% emotion) to 100% emotion for four emotions: happy, sad, angry, and fear. Morphing faces were combined into one‐sec movies progressing from 0% to 100% emotional display. In control trials, movies comprised a dark oval superimposed on a light‐grey oval, with similar structural characteristics to the face stimuli and which subsequently morphed into a larger shape, approximating the movement of the morphed faces. There were three blocks for each of the four emotional conditions with 12 stimuli per block, and six control blocks with six stimuli per block. Emotional and control blocks were presented in a pseudorandomized order so that no two blocks of any condition were presented sequentially. Participants were asked to use one of three fingers to press a button indicating the color of a semi‐transparent foreground color flash (orange, blue, or yellow) that appeared during the mid‐200–650 msec of the one‐sec presentation of the dynamically changing face.

fMRI Image acquisition

Functional neuroimaging data were collected at the University of Pittsburgh using a 3.0 Tesla Siemens Prisma MRI. Blood oxygenation level dependent (BOLD) images were acquired with a multi-band gradient echo EPI sequence (18 slices, threefactor multiband; 2.3 mm isotropic voxels; TR=1500ms, TE=30ms; field of view=220 × 220 mm; matrix 96 × 96; flip angle 55°, bandwidth 1860 Hz Px–1). Structural 3D axial MPRAGE images (TR=1500ms, TE=3.19ms; flip angle 8° FOV=256 × 256 mm; 1 mm isotropic voxels; 176 continuous slices) and fieldmaps (2.3 mm isotropic voxels; TR=500 ms, TE1=4.92 ms, TE2=7.38 ms; FOV=220 × 220 mm; flip angle 45°, bandwidth 1302 Hz Px–1) were acquired in the same session.

Preprocessing

Imaging data were preprocessed using a SPM, FSL, and AFNI implement in NiPype. Standard preprocessing steps were applied including realignment and coregistration, normalization, despiking, and spatial smoothing. SPM8 was used to generate first-level fixed-effects general linear models for each participant. The model included regressors for the four emotion types (fear, angry, sad, happy), shapes, motion parameters as covariates of no interest, and a regressor controlling for physiologic noise derived from the mean signal of white matter and CSF. A high-pass frequency filter (256s) and autoregressive modeling (AR(1)) was also applied.

First level activation analysis

From the first level activation maps, statistical parametric maps were generated for each of the four emotion contrasts (fear>shape; happy>shape; anger>shape; and sad>shape) to identify regions of activity related to processing each of these emotions across the entire sample using one sample t-test in SPM12. This search was constrained to an anatomically derived mask comprising regions implicated in emotional processing: bilateral amygdala, vmPFC, vlPFC, and mPFC including ACC, created using the WFU Pickatlas(<https://www.nitrc.org/projects/wfu_pickatlas>). Results were thresholded at clusters reaching FWE corrected p<0.05, with minimum cluster size 30 voxels to identify regions related to task. For each significant cluster, we used SPM to perform a PCA of the responses of the member voxels and extracted the eigenvalue of the first principal component. These principle eigenvalue parameter estimates, can be used to represent cluster-, contrast-, and participant-specific neural activity^3^, and were used in subsequent second level models.

Educational completion definition

(1) Less than seventh grade

(2) Seventh to ninth grade

(3) Partial high school

(4) High school diploma or General Equivalency Degree (GED)

(5) Some college (at least one year)

(6) Technical school or Associates Degree

(7) College diploma (Bachelor's Degree)

(8) Graduate or Professional Degree

Specificity of Findings Results

To further determine the specificity of the significant relationship between neural measures and impulsivity/mania/hypomania found in main analyses, we examined post-hoc whether the remaining self-report and clinician rated symptom measures were associated with each of the neural measures. Post-hoc analyses of left amygdala to facial fear (associated with impulsivity at baseline in main analyses) found no new significant relationships but 95% CI for the remaining self-report and clinician rated measures overlapped with the betas from the main findings (Supplemental table 10). Post-hoc analyses of left vlPFC to facial anger (associated with mania/hypomania at baseline) found no new significant relationships but 95% CI for the remaining measures also overlapped with the betas from the main findings (Supplemental table 11). Post-hoc analyses of left amygdala-mPFC FC to facial fear (which was associated with impulsivity at baseline) found significant negative relationships with the remaining measures (95% CI of beta reported): MASQ-AD[-0.56, -0.20], YMRS[-0.47, -0.11], HAM-D[-0.54, -0.19], and HAM-A[-0.53, -0.18] (Supplemental table 12). Post-hoc analyses of left amygdala to facial sadness (which predicted impulsivity at 6 months) found no new significant relationships but 95% CI for the remaining measures also overlapped with the betas from the main findings (Supplemental table 13).

Supplemental References

1. Sackeim HA. The definition and meaning of treatment-resistant depression. J Clin Psychiatry. 2001;62 Suppl 16:10-17.
2. Davis JM, Chen N. Dose response and dose equivalence of antipsychotics. J Clin Psychopharmacol. 2004;24(2):192-208.
3. Sugiura M, Friston KJ, Willmes K, Shah NJ, Zilles K, Fink GR. Analysis of intersubject variability in activation: An application to the incidental episodic retrieval during recognition test. *Human Brain Mapping* 2007; **28**(1)**:** 49-58.

| Emotion | Region | x | y | z | k | t_peak_ | p_FWE_ |
| --- | --- | --- | --- | --- | --- | --- | --- |
| Anger | L Amygdala | 22 | -4 | -20 | 152 | 12.5 | 0.002 |
|  | R Amygdala | -22 | -4 | -22 | 146 | 11.1 | 0.002 |
|  | L vlPFC | -36 | 30 | -20 | 157 | 6.2 | 0.001 |
|  | L vmPFC | -4 | 50 | -20 | 64 | 5.4 | 0.007 |
| Fear | L Amygdala | 22 | -4 | -20 | 151 | 10.6 | 0.001 |
|  | R Amygdala | -20 | -4 | -20 | 140 | 9.2 | 0.001 |
|  | L vlPFC | -32 | 34 | -20 | 31 | 5.6 | 0.013 |
|  | L vmPFC | -2 | 56 | -18 | 98 | 5.0 | 0.003 |
| Sad | L Amygdala | -20 | -4 | -20 | 134 | 7.9 | 0.002 |
|  | R Amygdala | 20 | -4 | -20 | 144 | 7.7 | 0.002 |
|  | L vmPFC | -2 | 52 | -18 | 72 | 5.0 | 0.006 |
| Happy | L Amygdala | -20 | -6 | -20 | 94 | 6.4 | 0.005 |
|  | R Amygdala | 30 | -4 | -22 | 125 | 7.3 | 0.003 |

Supplemental Table 1. Coordinates and significance of ROIs active across the entire sample for each emotion vs shape.

Controlling for diagnosis and medication

|  | UPPSP-Total | MASQ-AD |
| --- | --- | --- |
| Gender  MDD  GAD  L Amygdala Fear | 0.1451 0.0747  0.0442 0.6811  0.1944 0.0749  0.2312 0.1542 | * |

Supplemental Table 2. Predicting self-report measures at baseline. Excluding subjects with ADHD or bipolar disorder and controlling for gender and MDD and GAD diagnosis. Beta value followed by p-value.

|  | UPPSP-Total | MASQ-AD |
| --- | --- | --- |
| Gender  L Amygdala Fear | 0.1081 0.2061  0.3284 0.0581 | * |

Supplemental Table 3. Predicting self-report measures at baseline. Removing participants taking medication. Beta value followed by p-value.

|  | Negative Urgency | Lack of Premeditation | Lack of Perseverance | Sensation Seeking | Positive Urgency |
| --- | --- | --- | --- | --- | --- |
| Gender  MDD  GAD  L Amygdala Fear | -0.0386 0.7401  0.1434 0.3553  **0.5642 0.0004**  **0.6069 0.0102** |  | 0.2515 0.0226  0.3176 0.0303  0.2030 0.1664  **0.6700 0.0026** |  |  |

Supplemental Table 4. Neural activity associated with impulsivity subscales at baseline. Excluding subjects with ADHD or bipolar disorder and controlling for gender and MDD and GAD diagnosis. Beta value followed by p-value.

|  | Negative Urgency | Lack of Premeditation | Lack of Perseverance | Sensation Seeking | Positive Urgency |
| --- | --- | --- | --- | --- | --- |
| Gender  L Amygdala Fear | -0.1572 (0.2558)  **0.8374 (0.0032)** |  | 0.2003 (0.0942)  **0.6528 (0.0074)** |  |  |

Supplemental Table 5. Neural activity associated with impulsivity subscales at baseline. Removing participants taking medication and controlling for gender. Beta value followed by p-value.

|  | YMRS |
| --- | --- |
| Education  MDD  GAD  L vlPFC Anger | -0.1242 0.2514  **1.2741 0.0002**  0.7511 0.0257  -1.1278 0.0522 |

Supplemental Table 6. Predicting YMRS at baseline. Excluding subjects with ADHD or bipolar disorder and controlling for education and MDD and GAD diagnosis. Beta value followed by p-value.

|  | YMRS |
| --- | --- |
| Education  L vlPFC Anger | -0.2818 (0.0854)  **-2.1215 (0.0159)** |

Supplemental Table 7. Predicting YMRS at baseline. Removing participants taking medication. Beta value followed by p-value.

|  | UPPS-P Total |
| --- | --- |
| MDD  GAD  Baseline Impulsivity  Left Amygdala Sad | 0.1746 0.1791  -0.0259 0.8584  **1.0047 0.0000**  0.4514 0.0368 |

Supplemental Table 8. Predicting 6-month UPPS-P total, covarying for baseline UPPS-P total, excluding subjects with ADHD or bipolar disorder and controlling for MDD and GAD diagnosis. Beta value followed by p-value.

|  | UPPS-P Total |
| --- | --- |
| Medication load change  Baseline Impulsivity  Left Amygdala Sad | -0.0142 0.8509  **1.0324 0.0000**  0.5116 0.0200 |

Supplemental Table 9. Predicting 6-month UPPS-P total, covarying for baseline UPPS-P total and controlling for medication load change. Beta value followed by p-value.

| **L amygdala to facial fear associated with baseline symptoms** | |
| --- | --- |
| **Clinical Measure** | **95% Confidence Interval of Beta Value** |
| UPPS-P Total | **[0.02, 0.39]** |
| Negative Urgency | **[0.09, 0.46]** |
| Lack of Premeditation | [-0.05, 0.33] |
| Lack of Perseverance | **[0.09, 0.45]** |
| Sensation Seeking | [-0.24, 0.13] |
| Positive Urgency | [-0.09, 0.28] |
| MASQ-AD | [-0.002, 0.38] |
| YMRS | [-0.06, 0.32] |
| HAM-D | [-0.07, 0.31] |
| HAM-A | [-0.06, 0.33] |

Supplemental table 10. Testing for specificity of relationship between left amgydala activity to facial fear and UPPS-P scores. Reported values are 95% confidence intervals of beta values of left amygdala activity to facial fear predicting each other clinical measure, controlling for gender. Regular linear regression with zscored variables.

| **L vlPFC to facial anger associated with baseline symptoms** | |
| --- | --- |
| **Clinical Measure** | **95% Confidence Interval of Beta Value** |
| UPPS-P Total | [-0.26, 0.12] |
| Negative Urgency | [-0.21, 0.16] |
| Lack of Premeditation | [-0.23, 0.14] |
| Lack of Perseverance | [-0.24, 0.14] |
| Sensation Seeking | [-0.19, 0.18] |
| Positive Urgency | [-0.29, 0.08] |
| MASQ-AD | [-0.13, 0.25] |
| YMRS | **[-0.38, -0.02]** |
| HAM-D | [-0.23, 0.14] |
| HAM-A | [-0.21, 0.17] |

Supplemental table 11. Testing for specificity of relationship between left vlPFC activity to facial anger and YMRS scores. Reported values are 95% confidence intervals of beta values of left vlPFC activity to facial anger predicting each other clinical measure, controlling for education. Regular linear regression with zscored variables.

| **L Amygdala to mPFC FC during facial fear associated with baseline symptoms** | |
| --- | --- |
| **Clinical Measure** | **95% Confidence Interval of Beta Value** |
| UPPS-P Total | **[-0.44, -0.07]** |
| Negative Urgency | **[-0.49, -0.12]** |
| Lack of Premeditation | **[-0.43, -0.06]** |
| Lack of Perseverance | **[-0.58, -0.23]** |
| Sensation Seeking | [-0.01, 0.36] |
| Positive Urgency | [-0.34, 0.03] |
| MASQ-AD | **[-0.56, -0.20]** |
| YMRS | **[-0.47, -0.11]** |
| HAM-D | **[-0.54, -0.19]** |
| HAM-A | **[-0.53, -0.18]** |

Supplemental table 12. Testing for specificity of relationship between amygdala-mPFC FC during facial fear and UPPS-P. Reported values are 95% confidence intervals of beta values of brain measure predicting each other clinical measure, controlling for age, gender, and education. Regular linear regression with zscored variables.

| **L Amygdala to sadness predicting 6 month symptoms** | |
| --- | --- |
| **Clinical Measure** | **95% Confidence Interval of Beta Value** |
| UPPS-P Total | **[0.06, 0.48]** |
| Negative Urgency | **[0.05, 0.54]** |
| Lack of Premeditation | [-0.23, 0.31] |
| Lack of Perseverance | [-0.05, 0.38] |
| Sensation Seeking | [-0.06, 0.31] |
| Positive Urgency | [-0.10, 0.37] |
| MASQ-AD | [-0.07, 0.50] |
| YMRS | [-0.19, 0.65] |
| HAM-D | [-0.34, 0.30] |
| HAM-A | [-0.14, 0.50] |

Supplemental table 13. Testing for specificity of relationship between left amgydala activity to facial sadness and prediction of UPPS-P 6 months later. Reported values are 95% confidence intervals of beta values of left amygdala activity to facial sadness predicting each other clinical measure, controlling for respective baseline symptom severity. Regular linear regression with zscored variables.
